# Progress in clinical research and applications of retinal vessel quantification technology based on fundus imaging

**DOI:** 10.3389/fbioe.2024.1329263

**Published:** 2024-02-22

**Authors:** Naimei Chen, Zhentao Zhu, Weihua Yang, Qiang Wang

**Affiliations:** ^1^ Department of Ophthalmology, Huaian Hospital of Huaian City, Huaian, China; ^2^ Department of Ophthalmology, Shenzhen Eye Hospital, Jinan University, Shenzhen, China; ^3^ Department of Ophthalmology, Third Affiliated Hospital, Wenzhou Medical University, Ruian, China

**Keywords:** retinal vasculature, artificial intelligence, ocular diseases, systemic diseases, retinal vessel quantification

## Abstract

Retinal blood vessels are the only directly observed blood vessels in the body; changes in them can help effective assess the occurrence and development of ocular and systemic diseases. The specificity and efficiency of retinal vessel quantification technology has improved with the advancement of retinal imaging technologies and artificial intelligence (AI) algorithms; it has garnered attention in clinical research and applications for the diagnosis and treatment of common eye and related systemic diseases. A few articles have reviewed this topic; however, a summary of recent research progress in the field is still needed. This article aimed to provide a comprehensive review of the research and applications of retinal vessel quantification technology in ocular and systemic diseases, which could update clinicians and researchers on the recent progress in this field.

## 1 Introduction

Recent advancements in ophthalmic imaging technology have led to challenges in monitoring disease progression during early and advanced stages, largely due to its limited use for quantitative analysis of diseases; it has been mainly used for qualitative analysis of diseases. The rise in computational power, imaging data expansion, and ethical system improvement has led to a rapid development of AI in medicine (Ting et al., 2021). The retinal vessel quantification technology has been advanced, providing new opportunities for quantitative analysis of diseases. The retinal blood vessels are the only blood vessels that can be directly observed without using an invasive approach; their structures and functions are similar to those of the systemic vascular system. Quantitative research on retinal blood vessels could provide insights into the conditions of cardiovascular, cerebrovascular, and systemic blood vessels because they are components of the body’s circulatory system (Dumitrascu and Koronyo-Hamaoui, 2020). The AI-based retinal vessel quantification technology can help clinicians and researchers study ocular and systemic diseases, benefiting disease prevention, diagnosis, and treatment (Gadde et al., 2016; Fuchs et al., 2022; Fu et al., 2023). A few articles have reviewed this technology; however, summarizing its recent progress is still needed. This article aimed to provide a comprehensive review of the research and applications of retinal vessel quantification technology in ocular and systemic diseases. As shown in Table 1.

**TABLE 1:** Summary of key points of the article.

Retinal vessel quantification has become a hot area of research for common eye and related systemic diseases due to its high specificity and sensitivity
Clinical research and applications of retinal vessel quantification in eye diseases
Clinical research and applications of retinal vessel quantification in systemic diseases
Limitations and solutions in clinical research and applications of retinal vessel quantification
Researching and applying retinal vessel quantification to ocular and systemic diseases is promising

## 2 Retinal vessel quantification technology

Retinal vessel quantification is a method to measure and analyze retinal blood vessels in the fundus through imaging technology, and extensive research has been conducted in clinical Retinal vessel quantification based on AI using optical coherence tomography angiography (OCTA). OCTA utilizes specific algorithms to image only moving red blood cells in the vessels, providing three-dimensional (3D) visualization and quantitative analysis of the blood flow status across different vascular membrane layers of the retina and choroid (Lommatzsch, 2020; Koutsiaris et al., 2023). With the continual advancement in AI algorithms and ophthalmic imaging technology, several quantitative models for retinal blood vessels have been established, with high levels of sensitivity and specificity (Nguyen et al., 2013; Nunez do Rio et al., 2020; Comin et al., 2021; Liefers et al., 2021; Zhou et al., 2022; Nardini et al., 2023). Retinal vessel quantification is crucial in studying ocular and systemic diseases. Repeated quantitative analyses of vascular parameters such as vascular diameter, vascular fractal dimension (FD), vascular angle, vascular density (VD), retinal non-perfusion (RNP), and foveal avascular zone (FAZ) can assist in the comprehension of changes in disease occurrence, progression, and treatment (Pournaras and Riva, 2013; Lee et al., 2018; Ramos et al., 2019; Alibhai et al., 2020; Kadomoto et al., 2021).

Quantitative analysis of the normal retina allows for an in-depth examination of the retinal vascular structure, benefiting the diagnosis and analysis of vascular abnormalities. The density of the deep retinal vascular plexus is higher than that of the superficial retinal vascular plexus. The retina is divided into four sectors centered on the FAZ. The vascular density of the inferior region, whether deep or superficial, is higher than that of the other regions (temporal, superior, and nasal), and age has no significant effect on VD (Gadde et al., 2016). Macular vascular parameters are related to sex and age, and the FD and VD of blood vessels in males are significantly higher than those in females; in contrast, no significant differences exist in vessel curvature, and the entire macular region’s FD, VD, and average vascular diameter exhibit negative correlations with age (Feng et al., 2021). Based on the measurement of the retinal vascular parameters of individuals aged 50 and over, the greater the FAZ area and non-circular index, the lower the average capillary density and superficial vascular density, the poorer visual acuity. These vascular parameters might be used as biomarkers for predicting the visual acuity of those aged 50 and over (Li et al., 2023).

Retinal vessel quantification technology has the potential to foster research on relevant ailments and provide novel methods for managing systemic and ocular health. Precise quantitative analysis of the retinal vasculature can provide insight into the pathophysiological mechanisms of ocular diseases and early warning and monitoring of systemic diseases as shown in Table 2.

**TABLE 2:** The importance of quantifying retinal vessels in ocular and systemic diseases.

Ocular diseases	Systemic diseases
DR: Early diagnosis of disease, staging of disease severity, prediction of disease transformation, and assessment of treatment efficacy	Cerebrovascular diseases: Monitoring the risk of disease occurrence, alternative invasive or expensive examinations, and early diagnosis of diseases
RVO: Prediction of the risk of neovascularization and complications, selection of timing for PRP intervention, staging of disease severity, and evaluation of treatment effectiveness	Hypertension: Monitoring the risk of disease occurrence and predicting the risk of complications
Glaucoma: Detection of optic nerve injury, evaluation of visual function prognosis, and detection indicators of disease progression	CAD: Monitoring the risk of disease occurrence
EAMD: Detection indicators for disease progression	SCD: Monitoring the risk of disease occurrence
Other eye diseases: Selection of surgical methods and monitoring of disease progression	Fabry disease: Monitoring the risk of disease occurrence and predicting the risk of complications

## 3 Clinical research and applications in ocular disorders

### 3.1 Diabetic retinopathy

Diabetic retinopathy (DR) is a prevalent microvascular complication of diabetes and a frequent cause of blindness; the disease triggers vascular variations by damaging capillary cells like the vascular endothelial cells and pericytes, leading to local ischemia. DR progression leads to an increase in the number of microaneurysms (Wu et al., 2014; Jiang et al., 2020), vascular curvature (Lee et al., 2018), RNP area (Baxter et al., 2019; Alibhai et al., 2020; Kim et al., 2021), FAZ area (Mihailovic et al., 2019; Ratra et al., 2021; Meng et al., 2022), and non-circular index and a decrease in VD (Durbin et al., 2017). The RNP area shows a positive correlation with the emergence of neovascularization (Yu et al., 2020). These vascular parameters can function as reference indicators for the onset and progression of DR and predictive indicators for disease transformation. Certain parameters, including VD, vascular length, and FAZ area, could undergo changes prior to the onset of visual impairment in individuals with DR, and they offer substantial value in facilitating the detection of DR lesions at an early stage (Zhu et al., 2019). Among all parameters, there was a statistically significant difference in vascular curvature between patients with non-DR and mild non-proliferative diabetic retinopathy (NPDR), especially within the 1.5-mm area of the superficial layer of the retina (Lee et al., 2018). Quantitative indicators of retinal blood vessels can serve as potential biomarkers for DR staging (Xu et al., 2019; Chua et al., 2020; Boned-Murillo et al., 2021; Xu et al., 2021). Borrelli and colleagues attempted to utilize 3D analysis of OCTA to create a 3D image of the retinal vasculature and used a global threshold algorithm to procure two vascular parameters, 3D vascular volume, and 3D perfusion density, to assess the status of macular ischemia in patients with NPDR; the smaller the 3D vascular volume and 3D perfusion density, the more severe the macular ischemia (Borrelli et al., 2020). After thorough examinations, the reliability of 3D analysis for evaluating the state of retinal blood vessels has been confirmed, indicating broad potential applications (Borrelli et al., 2020). Retinal vessel quantification assesses the treatment prognosis of patients with DR, and it quantifies the alterations in retinal neovascularization pre- and post-treatment with anti-vascular endothelial growth factor (anti-VEGF) therapy to evaluate the susceptibility of patients with DR to VEGF (Hu et al., 2019); helping informed decisions for follow-on treatment. Pan-retinal photocoagulation (PRP) can effectively reverse retinal DR-caused ischemia while maintaining the integrity of macular microvascular structure. The treatment effect can be effectively evaluated by quantifying the retinal VD and FAZ areas after PRP in patients with DR (Abdelhalim et al., 2022; Sariyildiz et al., 2023).

### 3.2 Retinal vein occlusion

Retinal vein occlusion (RVO) is the second most common retinal vascular disease that can cause blindness following DR. It happens when one or more veins in the retina are blocked or obstructed. The retina, located at the posterior of the eye, detects light and transmits signals to the brain for visual perception. Venous obstruction interferes with normal blood flow on the retina, potentially resulting in vision impairments. The impact of RVO on the deep capillary plexus (DCP) of the retina is 1.77–1.84 times that of the shallow capillary plexus (SCP) (Kim et al., 2020). The larger the area of RNP, the greater the risk of neovascularization. Quantifying the area of RNP can effectively assess the risk of neovascularization (Kadomoto et al., 2021). Neovascularization and associated neovascular glaucoma are common complications of RVO that can cause serious damage to a patient’s vision and the eyeball itself (Hayreh, 2021). PRP can effectively prevent and treat neovascularization, which could benefit from quantifying the area of the RNP to select the timing of PRP use. The severity of macular ischemia increases as the VD in the macular area reduces and the RNP area expands, accurately quantifying the VD and the RNP area can be used to grade macular ischemia in RVO patients, and accurately quantifying the VD and the RNP areas can be used to grade macular ischemia in patients with RVO and assess disease severity and prognosis (Ouederni et al., 2019; Tang et al., 2021; Yeung et al., 2021). Huang et al., 2022 classified patients with branch vein occlusion (BRVO) into reactive and refractory groups based on their responses to anti-VEGF treatment, which was determined by semi-automatic quantitative fluorescein angiography to measure the amount of fluorescein leakage around and near the fovea of the macular area in patients with BRVO. The refractory group demonstrated more severe leakage than the reactive group; thus, this technique could effectively predict the efficacy of anti-VEGF treatment for evaluating treatment feasibility (Huang et al., 2022).

### 3.3 Glaucoma

Glaucoma is an irreversible condition that can cause blindness. Primary angle closure glaucoma is the most common type of glaucoma in China, which occurs when the angle between the iris and cornea in the front chamber of the eye becomes narrow or even closes completely, preventing the flow of aqueous humor. If the anterior chamber angle is narrow, the normal outflow of aqueous humor is impeded, causing an elevation in intraocular pressure, resulting in optic nerve damage and eventual vision loss. Based on the quantification of the retinal blood vessels in patients suffering from primary angle closure, the microvessel density around the optic disc reduces, despite the absence of any changes in the thickness of the retinal nerve fiber layer and the ganglion cell complex, the microvascular density around the optic papilla is a sensitive indicator of changes in intraocular pressure and a predictive and monitoring parameter for the onset of glaucoma nerve changes (Miguel et al., 2021; Nascimento E Silva et al., 2021; Wang et al., 2021). These observations highlight the potential utility of adjusting target intraocular pressure based on changes in microvessel density. Van Melkebeke et al., 2018 showed that the microvascular density surrounding the optic disc is a prognostic indicator of visual function in patients with glaucoma. Kromer et al., 2019 used OCTA to measure macular blood flow density in patients with open-angle glaucoma and found that macular blood flow density was significantly decreased in glaucoma patients compared to the healthy population. A noteworthy correlation exists between the density of blood flow in the macula and the visual field (Yarmohammadi et al., 2017), presenting an opportunity to repeatedly evaluate glaucoma progression by measuring macular blood flow density.

### 3.4 Wet age-related macular degeneration

Wet age-related macular degeneration (wAMD) is a common cause of irreversible visual impairment, typically affecting the central vision of the eye. It is usually caused by abnormal vascular growth beneath the retina, resulting in macular region damage and fluid exudation; the incidence rate of wAMD has increased significantly in recent years (Stahl, 2020). Gao et al. quantitatively analyzed the RNP areas of the extrafoveal superficial vascular complex (SVC), intermediate capillary plexus (ICP), and deep capillary plexus (DCP) in patients with wAMD and healthy individuals, ruling out related interfering factors. They found that these patients had larger areas of RNP in SVC, ICP, and DCP (Gao et al., 2022). Hence, it is evident that the area of the RNP is closely related to the onset and progression of wAMD; measuring the area of the RNP can be used in clinical practice to predict and monitor the onset and progression of wAMD.

Quantitative analysis of retinal effusion secondary to retinal angiopathy facilitates evaluating disease progression and treatment responses in patients with retinal effusion like RVO, wAMD, and diabetes macular edema (Farinha et al., 2020; Schmidt-Erfurth et al., 2020; Fuchs et al., 2022; Michl et al., 2022; Muste et al., 2022; Reiter and Schmidt-Erfurth, 2022; Coulibaly et al., 2023). Wu et al., 2021 proposed a new optimized segmentation and quantification algorithm for neovascularization based on OCTA, which has higher accuracy and effectively monitors changes in neovascularization. It can be useful in follow-up monitoring during diagnosing and treating ischemic ophthalmopathy and systemic diseases.

### 3.5 Other ocular diseases

Iris VD is decreased shortly after refractive surgery, and the densities of superficial and deep retinal blood vessels do not recover within 3 months of surgery (Olcay et al., 2015). The small incision corneal stromal lenticule extraction (SMILE) is more significantly reduced than femtosecond laser *in situ* keratomileusis (FS-LASIK), which might be related to an abnormal elevation in intraocular pressure during the surgical process (Olcay et al., 2015). Thus, it is necessary to consider the impact of vascular changes and the selection of surgical approaches for patients requiring refractive surgery in clinical practice (Cui et al., 2022). Retinal vessel quantification can monitor and analyze vascular changes in the occurrence, development, and treatment of eye diseases, such as retinitis pigmentosa (Wang et al., 2019; Lu et al., 2022), type 2 macular telangiectasia (Chidambara et al., 2016; Pauleikhoff et al., 2019; Pauleikhoff et al., 2022), familial retinal arteriolar tortuosity (Saraf et al., 2019), Behcet’s disease (Türkcü et al., 2020), optic disc drusen (Leal-González et al., 2020), and retinopathy of prematurity (Cabrera et al., 2021).

## 4 Clinical research and applications in systemic diseases

### 4.1 Cerebrovascular diseases

Retinal and cerebral blood vessels come from the internal carotid artery and interconnect and influence each other. Thus, abnormalities in retinal blood vessels are often accompanied by abnormalities in cerebral blood vessels, and abnormal changes in retinal blood vessels are associated with stroke, vascular cognitive impairment, and dementia (Frost et al., 2017; Cabrera DeBuc et al., 2018; Cabrera DeBuc et al., 2020; Dumitrascu and Koronyo-Hamaoui, 2020). Widespread retinal arteriolar stenosis is linked to a high risk of disabling dementia (Jinnouchi et al., 2017), which could serve as an effective biomarker for populations with disabling dementia and one of the traditional screening indicators. The APOE ε4 allele is among the genetic factors most closely linked with Alzheimer’s disease, and individuals carrying the APOE ε4 allele have a greater risk of developing Alzheimer’s disease. Carriers of the APOE ε4 allele have significantly higher vascular width ratios than normal individuals, and measuring the ratio of retinal vessel width is an alternative approach to the invasive examination of APOE ε4 (Frost et al., 2017). Brain white matter volume reduction and enlargement of the inferior lateral ventricle adversely affect brain function, potentially leading to cognitive impairments, motor dysfunction, epilepsy, and visual and speech issues, depending on the extent, location, and cause of the reduction. Brain white matter volume is typically assessed and evaluated using MRI, and a larger diameter of retinal venules is associated with a smaller brain white matter volume (Ikram et al., 2013). Decreased densities of retinal SCP and perfusion are associated with the enlargement of the inferior lateral ventricle (Yoon et al., 2019). As an alternative to MRI, assessing and monitoring changes in brain white matter volume and the inferior lateral ventricle can be achieved by measuring retinal venule diameter, retinal SCP density, and perfusion density. Early abnormalities in brain microvasculature, that cannot be detected by head MRI, are closely associated with changes in retinal vasculature. Therefore, quantifying retinal vascular changes can be used to assess brain vascular function, achieving early diagnosis and intervention (London et al., 2013; Wardlaw et al., 2013).

### 4.2 Hypertension

Hypertension is a common cardiovascular disease. As blood pressure becomes unstable or consistently rises, the risk of developing diseases, such as heart, retinal, stroke, and kidney diseases, also increases. Hypertension typically results in an elevated ratio of small artery length to diameter and decreased terminal branch arteries. Alterations in the retina often signal potential disease risks in other target organs; thus, quantifying retinal vascular parameters could help evaluate the risk of hypertension complications (Hughes et al., 2006; Leclaire et al., 2021). High blood pressure elevates the pressure on blood vessels, leading to weakened and hardened elasticity of retinal arterioles, compressing the veins, and resulting in decreased blood flow and gradual thinning of the veins, known as Gunn’s sign (Wigdahl et al., 2015). Furthermore, the pressure of arterioles at the intersection can be transmitted to the veins, resulting in the characteristic S-shaped appearance; the Salus sign is a term used to describe a phenomenon at the intersection of retinal arteries and veins, and quantifying the Gunn and Salus signs could predict hypertensive retinopathy and RVO development (Wigdahl et al., 2015). Bringing together deep learning and OCTA could more precisely quantify the retinal vascular structure, it is feasible to precisely forecast the onset and development risk of hypertension and its complications based on the changes in retinal vascular structure. (Tan et al., 2022).

### 4.3 Coronary artery disease

Coronary artery disease (CAD) is a cardiovascular disease occurring when the coronary artery (one of the main blood vessels supplying the heart) narrows or becomes blocked. CAD can trigger angina (chest pain) and myocardial infarction (severe damage to heart muscles), endangering the patient’s life. The high incidence and mortality rates of CAD imply early detection, and intervention are imperative. Fu et al. used deep learning technology to quantify retinal vascular parameters from color fundus photography of 57,947 participants without CAD; after approximately 11 years of follow-up, the FD of blood vessels had decreased, and the reduction in the number of arterial and small venous segments and arterial and venous bone densities was closely associated with an increased risk of subsequent CAD (Fu et al., 2023). Hence, detecting retinal vascular parameters could help predict CAD (Shokr et al., 2021).

### 4.4 Sickle cell disease

Sickle cell disease (SCD), also called sickle cell anemia, is a prevalent hereditary blood disease characterized by the deformation of red blood cells into sickle or curved shapes, different from the round shape of normal red blood cells. Such abnormal red blood cells are susceptible to adhesion and blockage within blood vessels, leading to ischemic damage. When OCTA was used to determine retinal capillary perfusion, dynamic changes were observed in retinal capillary perfusion in patients with SCD compared with healthy individuals (Zhou et al., 2021). Retinal ischemia and hypoxia can result in various complications. Non-invasive dynamic monitoring of retinal capillary perfusion in patients with SCD allows for effective evaluation of the state of systemic blood vessels, benefiting the early detection and treatment of the disease and assessing treatment efficacy.

### 4.5 Other systemic diseases

Fabry disease is a rare genetic disorder where glycolipids accumulate in multiple tissues and cells due to deficiency or reduced activity of the enzyme cleavage lipase, resulting in damage to multiple organs and systems. Quantifying retinal blood vessels demonstrate a negative correlation between retinal VD and myocardial damage associated with fabry disease (Cennamo et al., 2020). Primary nephrotic syndrome (PNS) is typically linked to dysfunction in glomerular filtration. The VD and blood flow perfusion density in the macular areas of patients with PNS significantly decreased compared with healthy individuals and negatively correlated with urinary protein levels (Yao et al., 2022). Thus, retinal blood vessels can be quantified to monitor and analyze the onset and progression of associated systemic illnesses and their complications and to assess disease alterations throughout treatment.

## 5 Limitations and solutions

Limitations exist with the retinal vessel quantification technology. This technology has limited generalization for large clinical applications, and trust and acceptance of algorithm outcomes vary among physicians and patients (Ting et al., 2019). Retinal vessel quantification techniques have some limitations, including inconsistent data quality, high equipment costs, inconsistent algorithms, and insufficient baseline data. To address these issues, we propose the following improvements: Firstly, we suggest improving reproducibility and reducing costs by enhancing imaging techniques and standardizing data acquisition and image processing processes. Secondly, we recommend developing international standards and guidelines to ensure algorithmic consistency and comparability. Thirdly, we propose establishing better baseline data through big data analytics to support clinical applications. Lastly, we suggest adopting data encryption and privacy protection measures to ensure the security of patient data. To enhance the acceptance of AI algorithms among physicians and patients, it is important to further develop explainable AI algorithms., Limitations and solutions of retinal vessel quantification are shown in Table 3. Fostering inter-team cooperation, improving technology, and increasing collaboration with clinicians and patients would help overcome these limitations, increasing its clinical applications and benefiting disease diagnosis and treatment.

**TABLE 3:** Limitations and solutions of retinal vessel quantification.

Limitations	Solutions
Data quality is inconsistent	Improving imaging technology, standardizing data acquisition and image processing, and improving repeatability
The equipment used for quantification is complex and expensive, which is not conducive to dissemination	Reducing the cost of equipment and promoting technology use in remote areas
The inconsistency of retinal vessel quantification algorithms leads to variable results	Developing international standards and guidelines to ensure consistency and comparability of retinal vessel quantification algorithms
The lack of sufficient baseline data for comparison with individual retinal vascular diseases may limit its clinical applications	By collecting large-scale retinal images and clinical data, baseline data can be better established; this will help improve disease diagnosis and treatment
Patient privacy and safety issues	Taking data encryption and privacy measures to ensure the security and legality of patient data
Doctors and patients have a low acceptance of AI “black box” algorithms	Further development of interpretable AI algorithms

## 6 Application trends and prospects

The development of modern technologies, dataset expansion, improvement in doctor-patient acceptance, data privacy and ethics, and increased financial support will render retinal vessel quantification to have broader application prospects in disease research. By observing changes in the retinal blood vessels of diverse populations, researchers evaluate the effectiveness of disease treatment protocols and study pathophysiologic changes in disease to more accurately diagnose and treat disease and develop drugs (Al-Shabrawey, 2023; Middel et al., 2023; Yucel Gencoglu et al., 2023). Prospects and trends of retinal vessel quantification in clinical disease are shown in Table 4.

**TABLE 4:** Prospects and trends of retinal vessel quantification in clinical disease.

Personalized medicine: As technology advances, the quantification of retinal blood vessels will become a part of personalized medicine. Doctors can develop personalized treatment plans based on the specific retinal characteristics of each patient to improve treatment efficacy
Predictive medicine: AI can help doctors predict eye and systemic health risks by analyzing large-scale retinal image data and cases. This predictive medicine helps with early intervention and disease prevention
New drug development: Retinal vessel quantification can be used to evaluate the therapeutic effects of new drugs on diseases, speeding up the development of new drugs and providing more treatment options for patients
Identification of disease subtypes: Vascular quantification can help identify subtypes of different diseases, leading to more accurate diagnosis and treatment
Remote monitoring: The remote acquisition and analysis of retinal images allow doctors to monitor patients regularly without frequent clinic visits. This is particularly beneficial for managing long-term chronic conditions
Multi-field applications in ocular and systemic diseases: Retinal vessel quantification has applications in ophthalmology and plays a role in various medical fields, such as systemic health management and cardiovascular disease research

Retinal vessel quantification will continue to play an important role in clinical research. It would improve the effectiveness of disease diagnosis, treatment, and prevention and promote the further development of medical science by combining advanced technologies and data analysis methods.

## 7 Conclusion

The quantification of retinal vessels has important clinical research and application values, mainly reflected in the following aspects. First, it can be used for early diagnosis of diseases. Doctors can detect early signs of DR, glaucoma, CAD, and other diseases by quantifying retinal vessels, allowing for early intervention and treatment to reduce permanent damage. Second, it can be used to monitor disease progression. Retinal vessel quantification can be used to monitor disease progression, determine the effectiveness of treatment, and adjust treatment plans in a timely manner in patients with eye and related systemic diseases. Third, it can be used for personalized treatment. By understanding each patient’s retinal vascular status, physicians can develop more personalized treatment plans to improve treatment efficacy and reduce side effects. Fourth, it can assist in replacing relevant clinical examinations. Retinal vessel quantification can effectively assist in replacing some traumatic and costly clinical examinations with repeatability, helping clinicians evaluate disease occurrence, development, and treatment effectiveness. Fifth, it can be used for studying disease mechanisms and pathogenesis, providing a foundation for new treatment methods and drug development.

Overall, retinal vessel quantification has broad research application prospects in the diagnosis, treatment, and research of ocular and systemic diseases, helping prevent disease onset and development and better protect and maintain the physical health of patients (Keskinbora and Güven, 2020).
